# Renal Peripelvic Extramedullary Haematopoiesis in Myelofibrosis: A Rare Imaging Pitfall Assessed by Multimodality Nuclear Imaging

**DOI:** 10.3390/diagnostics16071011

**Published:** 2026-03-27

**Authors:** Redouane Soussi, Ayoub Jaafari, Anas Chbabou, Sara Zouggari, Manar Zaiter, Tom Saliba, Patrick Flamen

**Affiliations:** 1 Nuclear Medicine Department, H.U.B Hospital, 1070 Brussels, Belgium; redouane.soussi@ulb.be (R.S.);; 2 Radiology Department, H.U.B Hospital, 1070 Brussels, Belgium

**Keywords:** extramedullary haematopoiesis, renal EMH, myelofibrosis, FDG-PET/CT, technetium-99m sulphur colloid, SPECT/CT, renal peripelvic infiltration

## Abstract

Extramedullary haematopoiesis (EMH) refers to haematopoietic proliferation outside the bone marrow, most often arising as a compensatory response to ineffective marrow function in chronic anaemias and myeloid neoplasms, particularly myelofibrosis and other myeloproliferative neoplasms. While the liver and spleen are typical sites, renal involvement remains particularly uncommon and may mimic infiltrative malignancy or infection on cross-sectional imaging. We report a 35-year-old woman with biopsy-proven grade 2 myelofibrosis who presented with constitutional symptoms, namely asthenia, progressive weight loss, and intermittent fever, in the setting of pancytopenia. Contrast-enhanced CT demonstrated bilateral thoracic paravertebral and presacral soft-tissue masses, with left peripelvic/pelvicalyceal infiltration, raising concern for infiltrative malignancy or infection. [^18^F]-FDG-PET/CT showed low-grade uptake in the paravertebral and presacral lesions, while the renal lesion remained indeterminate because of adjacent urinary tracer activity. Given the haemorrhagic risk of renal biopsy in a cytopenic patient, [^99m^Tc]-sulphur colloid scintigraphy with SPECT/CT was performed and demonstrated concordant tracer uptake in all lesions, supporting multifocal EMH. After disease-directed treatment, follow-up CT at 12 months showed marked regression of the renal and other EMH lesions. This case highlights renal peripelvic EMH as a rare imaging pitfall and underscores the value of multimodality imaging when biopsy is high risk.

**Figure 1:**
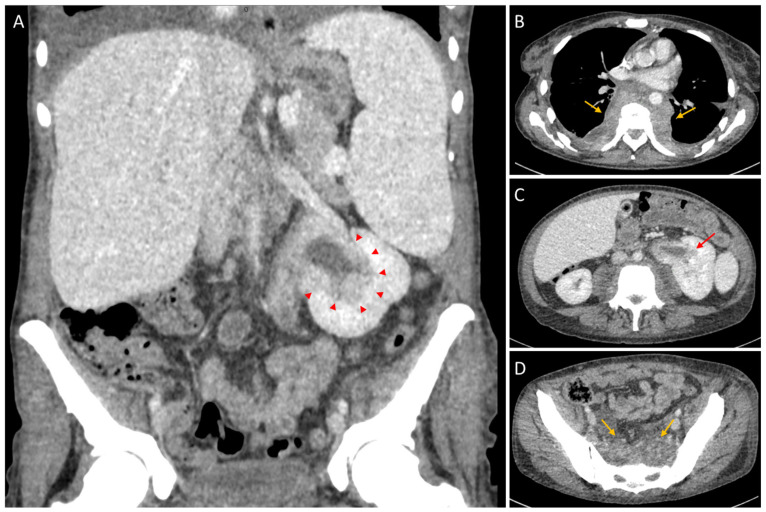
**Contrast-enhanced thoraco-abdomino-pelvic CT.** (**A**). Coronal reconstruction showing hepatomegaly and splenomegaly, with left peripelvic/pelvicalyceal soft-tissue infiltration (red arrowheads) associated with renal pelvic dilatation. (**B**). Axial chest CT demonstrating bilateral thoracic paravertebral soft-tissue masses (orange arrows). (**C**). Axial abdominal CT illustrating the left peripelvic/pelvicalyceal infiltrative abnormality (red arrow). (**D**). Axial pelvic CT showing a presacral soft-tissue lesion (orange arrows). A 35-year-old woman was referred to the haematology department for evaluation of a persistent systemic inflammatory syndrome associated with constitutional symptoms, including intermittent fever, drenching night sweats and unintentional weight loss of approximately 5–6 kg over 3 months. The symptoms had been present for around 6 months, with progressive worsening. Her medical history included iron deficiency anaemia on supplementation, poorly controlled type 2 diabetes mellitus, and well-controlled arterial hypertension. Laboratory investigations revealed severe pancytopenia with aregenerative anaemia (Hb 7.1 g/dL; reticulocytes 18 × 10^9^/L), leukopenia (WBC 1.2 × 10^9^/L) with profound neutropenia (0.45 × 10^9^/L), and thrombocytopenia (platelets 52 × 10^9^/L), together with marked inflammation (CRP 156 mg/L). Lactate dehydrogenase (LDH) was moderately elevated (520 U/L), while renal and liver function were preserved (creatinine 0.92 mg/dL; AST 31 U/L; ALT 34 U/L). Peripheral blood smear showed 5% circulating blasts. First-line infectious and parasitological investigations were negative, including blood cultures, HIV and hepatitis B/C serology, CMV and EBV IgM, and malaria testing (rapid antigen test and thick film). Given the cytopenias, bone marrow aspirate and trephine biopsy showed a hypercellular marrow with dysplastic features and megakaryocytic atypia, without CD34-defined blast excess; reticulin/Masson trichrome staining confirmed grade 2 myelofibrosis with focal collagen deposition.

**Figure 2:**
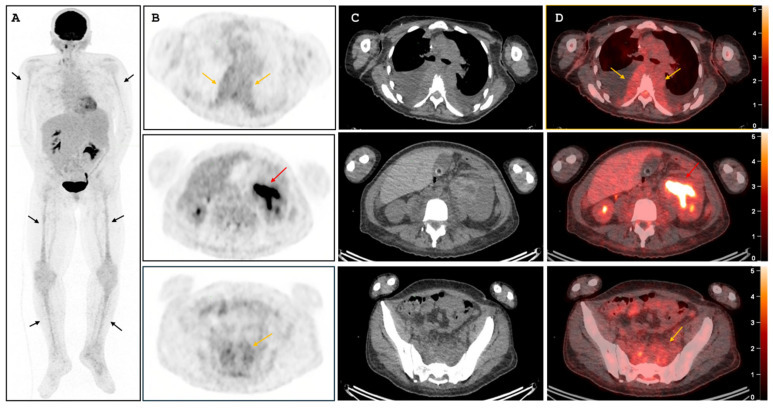
**[^18^F]-FDG-PET/CT**. (**A**) Maximum intensity projection (MIP) showing diffuse heterogeneous skeletal uptake, predominantly involving the long bones (black arrows), in keeping with peripheral redistribution of active haematopoiesis. (**B**) Axial PET images demonstrating low-grade uptake in the thoracic paravertebral masses (orange arrows, upper panel) and presacral lesion (orange arrow, lower panel). Evaluation of the left renal peripelvic/pelvicalyceal lesion (red arrow, middle panel) is limited by adjacent physiological urinary tracer activity. (**C**) Corresponding axial low-dose CT images. (**D**) Corresponding fused PET/CT images. To further characterize the CT abnormalities, [^18^F]-FDG-PET/CT was performed. It showed diffuse heterogeneous skeletal uptake, predominantly involving the long bones, suggesting redistribution of active haematopoiesis to the peripheral skeleton. The thoracic paravertebral and presacral soft-tissue lesions demonstrated only low-grade uptake, whereas the left peripelvic/pelvicalyceal lesion remained metabolically indeterminate because evaluation was limited by adjacent physiological urinary activity. This renal lesion raised an important diagnostic dilemma between upper tract urothelial malignancy and extramedullary haematopoiesis related to the underlying haematological disorder.

**Figure 3:**
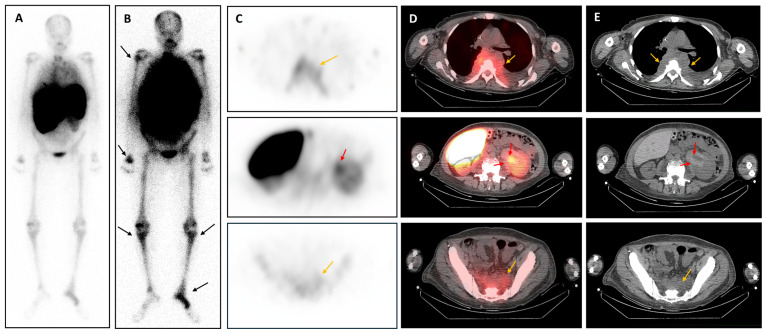
**[^99^mTc]-sulphur colloid scintigraphy with SPECT/CT.** (**A**) Whole-body planar anterior image. (**B**) Corresponding anterior planar image displayed with increased intensity scaling to better depict the heterogeneous skeletal tracer distribution, most evident in the peripheral skeleton and long bones (black arrows), consistent with redistributed active haematopoiesis. (**C**) Axial SPECT images showing tracer uptake in the thoracic paravertebral lesion (orange arrow, upper panel), left peripelvic/pelvicalyceal lesion (red arrow, middle panel), and presacral lesion (orange arrow, lower panel). (**D**) Corresponding fused SPECT/CT images. (**E**) Corresponding axial CT images. As tissue sampling was considered unsafe because of the high haemorrhagic risk in this cytopenic patient, [^99m^Tc]-sulphur colloid scintigraphy with SPECT/CT was performed to support a marrow-related aetiology (Figure 3). This demonstrated concordant tracer uptake in the paravertebral, presacral, and left peripelvic lesions, strongly supporting multifocal extramedullary haematopoiesis (EMH). Initial management consisted of supportive care, including platelet transfusions. She was then treated with ruxolitinib for 3 months as bridging therapy before allogeneic haematopoietic stem cell transplantation, with subsequent favourable clinical and biological improvement over the following 6–8 months. Extramedullary haematopoiesis (EMH) is a compensatory process in which haematopoiesis occurs outside the bone marrow when normal marrow function becomes ineffective, most commonly in chronic anaemias and myeloid neoplasms such as myelofibrosis [1,2]. Although previously described, renal EMH remains rare, and its imaging appearance may still pose a significant diagnostic challenge [1,2,3,4,5,6,7]. Reported CT appearances of renal EMH include perinephric plaques, hilar or perihilar masses, and infiltrative soft tissue centred on the renal sinus and pelvicalyceal system, sometimes extending to the proximal ureter and causing obstruction or renal dysfunction [1,2,3,4,5,6,7]. In our patient, the principal differential diagnoses were upper tract urothelial carcinoma, infiltrative haematological disease such as lymphoma or leukaemic/myeloid infiltration, and, less likely, other infiltrative inflammatory or infectious processes [3,4,5,6,7].

**Figure 4:**
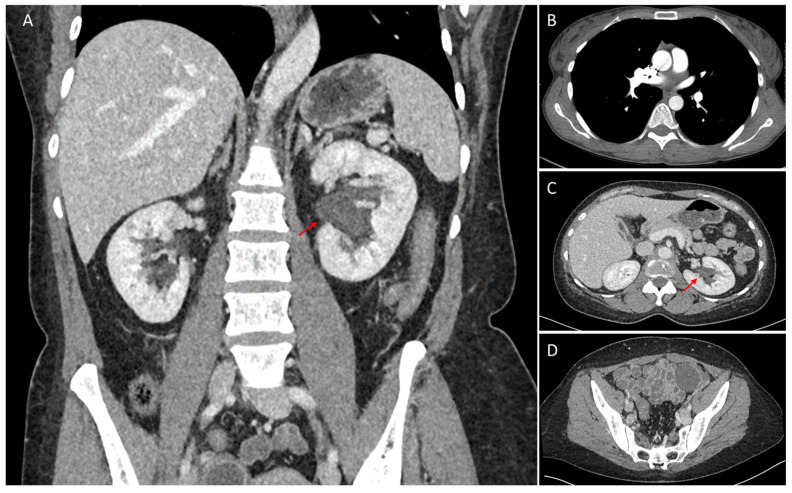
Follow-up contrast-enhanced thoraco-abdomino-pelvic CT performed 12 months after treatment. (**A**) Coronal reconstruction showing marked resolution of the left peripelvic/pelvicalyceal soft-tissue infiltration (red arrow), compared with baseline imaging. (**B**) Axial chest CT showing complete resolution of the thoracic paravertebral lesions. (**C**) Axial abdominal CT showing marked resolution of the left peripelvic/pelvicalyceal infiltrative lesion (red arrow). (**D**) Axial pelvic CT showing complete resolution of the presacral lesion. Reticuloendothelial- or marrow-targeted radiotracers can provide direct functional evidence of ectopic haematopoietic tissue [2,8]. This was particularly relevant in our patient because [^18^F]-FDG-PET/CT remained non-specific [2,9,10]. In this setting, [^99m^Tc]-sulphur colloid SPECT/CT may support the diagnosis of EMH by demonstrating uptake at ectopic sites, while hybrid imaging improves anatomical localization [2,8,10]. It has also been used in primary myelofibrosis to assess marrow distribution and identify sites of extramedullary haematopoiesis [11]. In our patient, concordant uptake in the paravertebral, presacral, and renal lesions strongly supported multifocal EMH. This multimodality approach was clinically relevant because biopsy of the renal lesion was considered unsafe in the setting of marked pancytopenia and anticipated haemorrhagic risk [3,4,5,6,7]. Initial management consisted of supportive care, including platelet transfusions, followed by a 3-month course of ruxolitinib as bridging therapy before allogeneic haematopoietic stem cell transplantation [12,13]. Favourable clinical and biological improvement was observed over the following 6–8 months, and follow-up contrast-enhanced abdominal CT at 12 months showed marked resolution of the left renal peripelvic infiltration together with regression of the other EMH sites (Figure 4). This case has important limitations. Histopathological confirmation of the renal lesion was not obtained because biopsy was considered unsafe; the diagnosis therefore relied on the concordant multimodality imaging pattern and subsequent radiological regression after treatment. In conclusion, renal peripelvic EMH is an uncommon but important imaging pitfall that may closely mimic infiltrative malignancy on CT. In cytopenic patients in whom biopsy is unsafe or high risk, [^18^F]-FDG-PET/CT and [^99m^Tc]-sulphur colloid SPECT/CT, together with follow-up imaging, can provide strong non-invasive diagnostic support and help guide management.

## Data Availability

The data presented in this study are available on request from the corresponding author due to privacy.
